# HORNET: tools to find genes with causal evidence and their regulatory networks using eQTLs

**DOI:** 10.1093/bioadv/vbaf068

**Published:** 2025-04-18

**Authors:** Noah Lorincz-Comi, Yihe Yang, Jayakrishnan Ajayakumar, Makaela Mews, Valentina Bermudez, William Bush, Xiaofeng Zhu

**Affiliations:** Case Western Reserve University Department of Population and Quantitative Health Sciences, Cleveland, OH 44106, United States; Case Western Reserve University Department of Population and Quantitative Health Sciences, Cleveland, OH 44106, United States; Case Western Reserve University Department of Population and Quantitative Health Sciences, Cleveland, OH 44106, United States; Case Western Reserve University Department of Population and Quantitative Health Sciences, Cleveland, OH 44106, United States; Case Western Reserve University Department of Neurosciences, Cleveland, OH 44106, United States; Case Western Reserve University Department of Population and Quantitative Health Sciences, Cleveland, OH 44106, United States; Case Western Reserve University Department of Population and Quantitative Health Sciences, Cleveland, OH 44106, United States

## Abstract

**Motivation:**

Nearly two decades of genome-wide association studies (GWAS) have identify thousands of disease-associated genetic variants, but very few genes with evidence of causality. Recent methodological advances demonstrate that Mendelian randomization (MR) using expression quantitative loci (eQTLs) as instrumental variables can detect potential causal genes. However, existing MR approaches are not well suited to handle the complexity of eQTL GWAS data structure and so they are subject to bias, inflation, and incorrect inference.

**Results:**

We present a whole-genome regulatory network analysis tool (HORNET), which is a comprehensive set of statistical and computational tools to perform genome-wide searches for causal genes using summary level GWAS data, i.e. robust to biases from multiple sources. Applying HORNET to schizophrenia, eQTL effects in the cerebellum were spread throughout the genome, and in the cortex were more localized to select loci.

**Availability and implementation:**

Freely available at https://github.com/noahlorinczcomi/HORNET or Mac, Windows, and Linux users.

## 1 Introduction

Genetic epidemiologists have spent decades trying to identify genes that cause disease (Hormozdiari *et al.* 2015). Significant effort has been given to experimental methods (Rees and Alcolado 2005, Tai *et al.* 2011), linkage studies (Ott *et al.* 2015), genome-wide association studies (GWAS), and functional annotation of putative disease-associated genetic variants (Sullivan *et al.* 2023). These methods of causal validation may be costly, may not always provide causal inference, and have sometimes produced conflicting results (Lewandowski *et al.* 2020). They also generally cannot be scaled to efficiently test hundreds or thousands of genes simultaneously. Cis-Mendelian randomization (cisMR) has been proposed as a cost- and time-efficient alternative to identify potential causal genes and can leverage the wealth of publicly available summary data from GWAS and eQTL studies (Porcu *et al.* 2019, van Der Graaf *et al.* 2020, Gleason *et al.* 2021, Zhu *et al.* 2021a). In this context, cisMR uses instrumental variables that are gene expression quantitative trait loci (eQTLs) to estimate tissue-specific causal effects of gene expression on disease risk (Gill *et al.* 2021).

cisMR methods are similar to transcriptome-wide association study (TWAS) methods, which test the association between predicted gene expression and the outcome phenotype. TWAS may suffer from reduced power due to imprecise estimation of gene expression in the discovery population (van Iterson *et al.* 2017, Dai *et al.* 2023, Liang *et al.* 2023), and from direct SNP associations with the outcome phenotype, known as horizontal pleiotropy. MR requires only GWAS summary statistics and a range of robust tools to control Type I error and bias from horizontal pleiotropy rate have been developed (Jiang *et al.* 2022, Lorincz-Comi *et al.* 2024). The MR-based approach can either consider each gene separately (univariable MR) or jointly with surrounding genes in a regulatory network (multivariable MR). Since it is well known that many genes are members of large regulatory networks (Karlebach and Shamir 2008, Emmert-Streib *et al.* 2014), multivariable MR may be better suited to study multiple gene expressions simultaneously than univariable MR that study one gene expression and one trait separately, such as TWAS (Sanderson 2020, Lin *et al.* 2023, Lorincz-Comi *et al.* 2024).

However, there is currently no unified statistical or computational framework for applying multivariable MR to the study of causal genes. Performing multivariable MR with summary data from eQTL and disease GWAS (eQTL-MVMR) has many challenges, including the handling of missing data, linkage disequilibrium (LD) between eQTLs, gene tissue specification, gene prioritization, and causal inference. Without careful attention to each of these challenges, the simple application of traditional multivariable MR methods to these data may produce spurious results which may fail in follow-up experimental testing. We present HORNET, a set of bioinformatic tools that can be used to robustly perform eQTL-MVMR with GWAS summary data. We demonstrate that existing univariable and multivariable implementations of eQTL-MR are vulnerable to biases and/or inflated Type I and II error rates from weak eQTLs, correlated horizontal pleiotropy (CHP), high correlations between genes, missing data, and mis-specified LD structure.

## 2 Methods

### 2.1 Data

**Figure 1.**
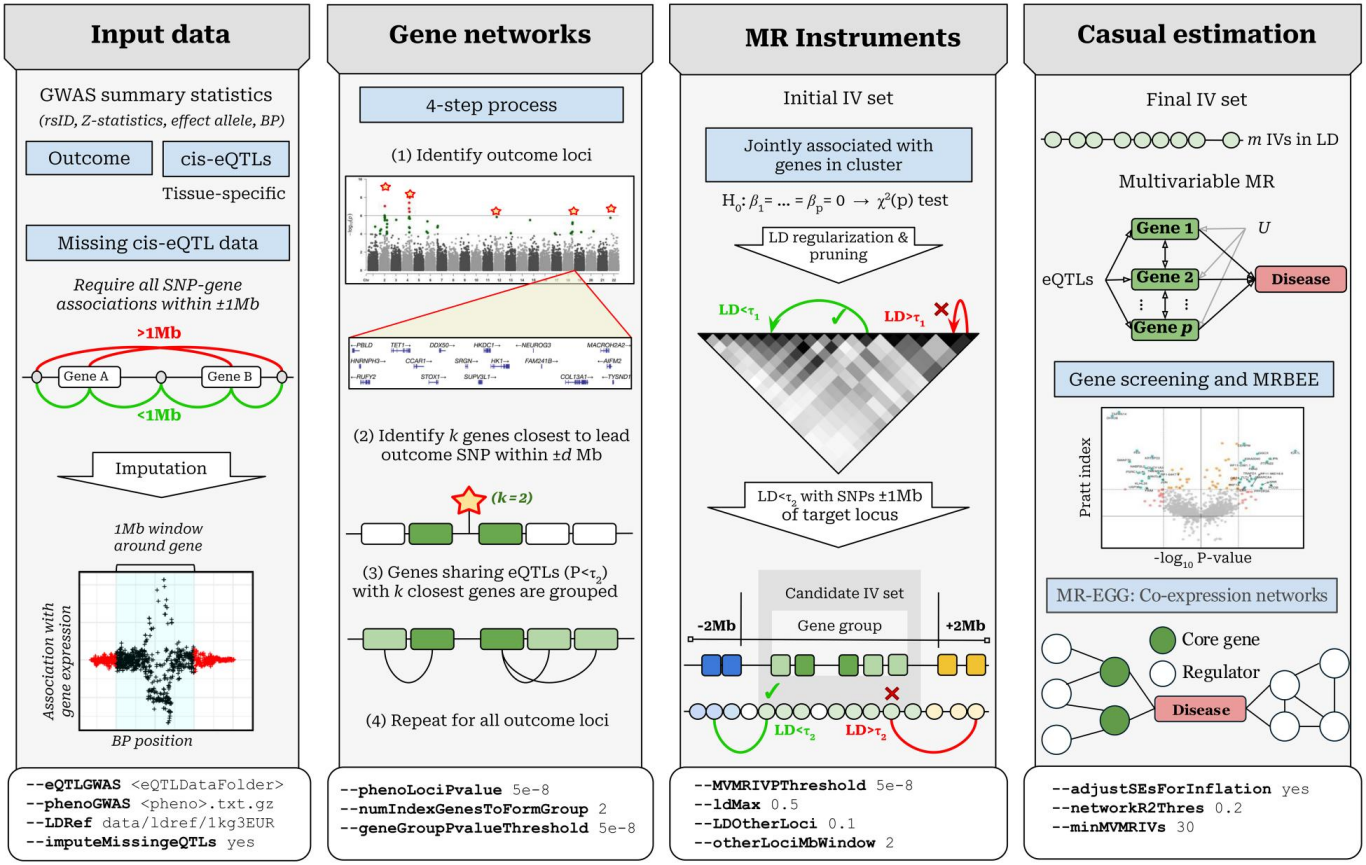
Flowchart illustrating genome-wide causal gene searches using HORNET. Example options given to flags that the command line version of HORNET uses are at the bottom of each panel. In the “Input data” section, ±1 Mb is used because it is standard in many publicly available data such as GTEx (2015) and eQTLGen (Vosa *et al.* 2021). The HORNET software is available from https://github.com/noahlorinczcomi/HORNET.

HORNET uses summary level data from GWAS of cis gene expression (eQTL) and a disease phenotype. cis-eQTL GWAS data should generally provide estimates of association between the expression of each gene and all SNPs within ±1 Mb of them (see Fig. 1). These data are publicly available from consortia such as eQTLGen (Vosa *et al.* 2018), the Genotype-Tissue Expression (GTEx) project (2015), and many others. Disease GWAS data can typically be downloaded from public repositories such as the GWAS Catalog (Sollis *et al.* 2023). HORNET additionally requires an LD reference panel with corresponding .bim, .bed, and .fam files. The 1000 Genomes Phase 3 (1 kg) (The 1000 Genomes Project Consortium *et al.* 2015) reference panel is automatically included with the HORNET software for African, East Asian, South Asian, European, Hispanic, and trans ancestry populations, although researchers may use their own reference panels such as those from the UK Biobank (Sudlow *et al.* 2015).

### 2.2 Instrument selection and missing data

Selection of the IV set in eQTL-MVMR using standard IV selection methods can either reduce statistical power or make estimation of causal effects impossible because of the structure of cis-eQTL GWAS summary statistics. Univariable eQTL-MR for the *k*th gene in a locus of *p* genes uses the set $S_{k}$ of cis-eQTLs as IVs and performs univariable regression (Gkatzionis *et al.* 2023). Multivariable eQTL-MR in the same locus uses the superset $S_{\cup }=\cup_{k=1}^{p}S_{k}$ and performs multivariable regression (Porcu *et al.* 2019). Since most publicly available cis-eQTL data only contain estimates of association between SNPs and all genes within *±*1 Mb of them (e.g. Vosa *et al.* 2018), not all SNPs in $S_{\cup }$ may have association estimates that are present in the data. An alternative approach is to use the set $S_{\cap }=\cap_{k=1}^{p}S_{k}$ which contains SNPs with association estimates that are available for all *p* genes. However, this set may contain very few SNPs, if any, for some relatively large loci which contain many genes that are co-regulated. If the size of $S_{\cap }$ is small, there can be limited statistical power for eQTL-MVMR because the power in MR is proportional to the total trait variance explained by the IVs (Lorincz-Comi *et al.* 2024). Thus, only $S_{\cup }$ is used in HORNET.

**Figure 2.**
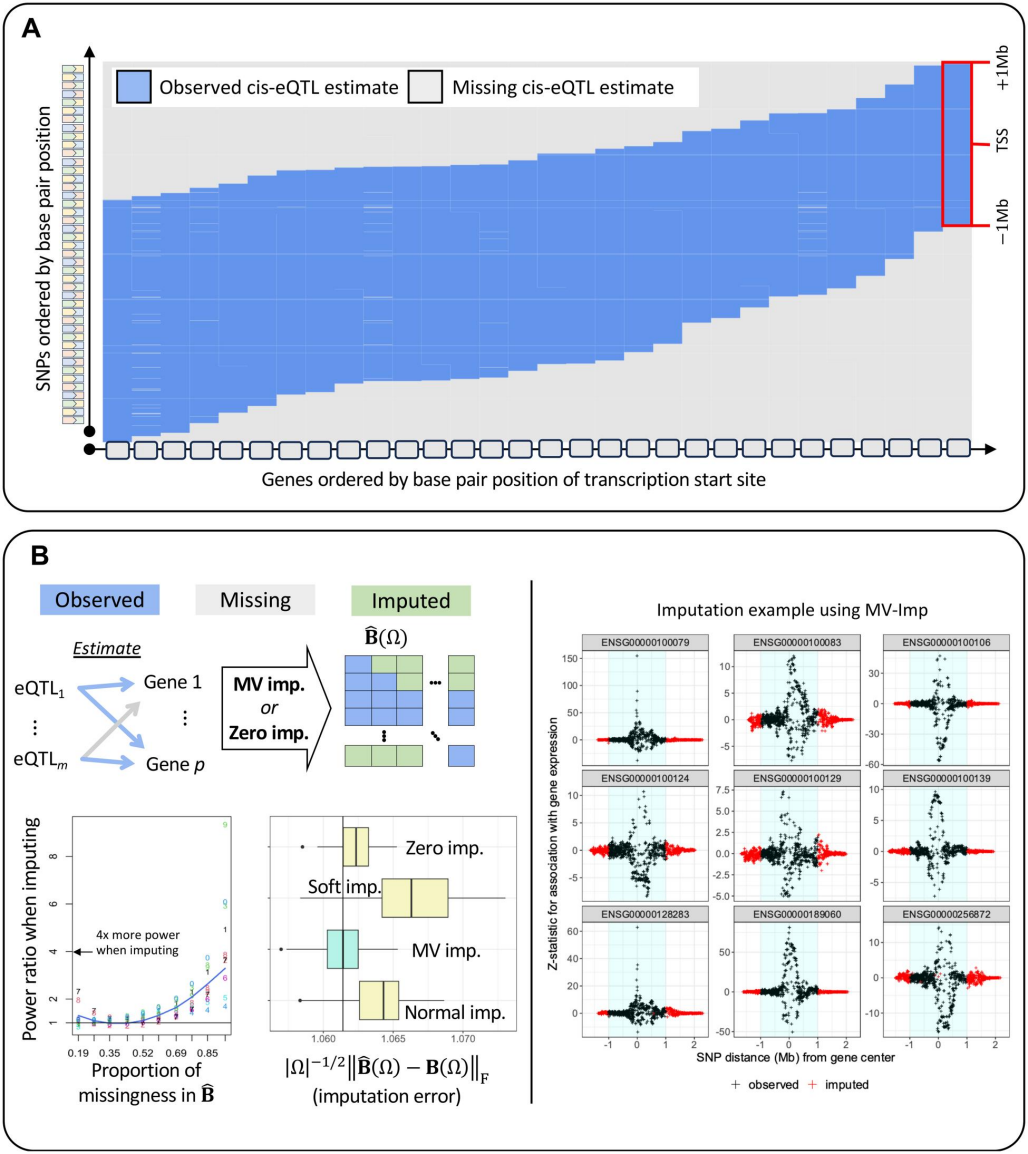
This figure illustrates the mechanism in summary cis-eQTL GWAS data that leads to missing data in eQTL-MVMR and how this missing data can be addressed using imputation. (A) Only SNP-gene pairs within a defined distance have association estimates present in cis-eQTL summary data. This figure demonstrates this by displaying the available data for SNPs and genes ordered by their chromosomal position using data from the eQTLGen Consortium (Vosa *et al.* 2018). (B) (left) Visual display of the pattern of missing in the design matrix $\hat{B}(\Omega )$ used in eQTL-MVMR. Imputation can be performed by setting missing values to be 0 (“Zero imp.”) or by applying the low-rank approximation (“MV imp.”) to $\hat{B}(\Omega )$ described in Algorithm 1. “Soft impute” is the soft imputation method of Hastie *et al.* (2015) and “Normal imp.” is a gene-pairwise imputation method based on the multivariate normal distribution, more fully described in the Supplementary material. |Ω| is the total number of missing values in a simulation of 1000 replicates performed using real data in the *CCDC163* gene region. These data were GWAS summary statistics of gene expression in blood tissue measured in 236 unrelated non-Hispanic White individuals. Full details of this simulation are presented in the Supplementary material. (right) An example of the MV imp. method applied to summary data for nine genes on chromosome 22 using cis-eQTL data from the eQTLGen Consortium (Vosa *et al.* 2018).

We propose imputing missing data using one of three approaches that users of HORNET can choose between: (i) imputation of missing values with 0 s, (ii) imputation based only on LD structure between observed and unobserved SNPs (Rueger *et al.* 2018), and (iii) imputation based on a modified matrix completion algorithm (MV-Imp). Using any of these methods, only estimates of association between SNPs and the gene expression phenotype are imputed. The MV-Imp approach in (iii) is applied to SNPs in the union set $S_{\cup }$ presented in Algorithm 1. This approach assumes a low-rank structure of the MR design matrix and accounts for estimation error and LD structure. As mentioned, public cis-eQTL summary data are generally available for SNP-gene pairs within *±*1 Mb of each other. Using individual-level data from 236 unrelated non-Hispanic White subjects, we demonstrate in Supplementary Fig. S4 that association estimates outside of the 1 Mb window have mean 0 and constant variance with high probability. Imputation using MV-Imp imputes data with the lowest error in simulation compared to existing approaches (see Fig. 2), though imputation of missing values with zeros performs similarly and is more computationally efficient. Figure 2 also demonstrates that imputation of missing eQTL association estimates generally leads to increased statistical power for testing the causal null hypothesis in MVMR compared to excluding eQTLs with missingness for any tested gene. Nevertheless, users of HORNET have the option to impute missing eQTL association estimates with all zeros or using the procedure in Algorithm 1. Results presented in Supplementary Section S6 using real data show that imputation with zeros can in some cases lead to better fitting models, though on average imputation using the algorithm in Algorithm 1 does.

After imputing the missing SNP-expression association estimates, the full set of candidate IVs $S_{\cup }$ is restricted to those that are significant in a joint test of association. Let ${\hat{\beta }}_{j}$ be the *p*-length vector of associations between the *j*th eQTL in $S_{\cup }$ and the expression of *p* genes in a tissue, where $\mathrm{Cov}({\hat{\beta }}_{j})≔\Sigma$ is estimated using the insignificant eQTL effect estimates method (Lorincz-Comi *et al.* 2024). The initial candidate set $S_{\cup }$ is restricted to


(1) S={j: β^j⊤Σ-1β^j>Fχ2(p)-1(1-α)}

where $\alpha =5\times 10^{-8}$ by default in the HORNET software. The set S is further restricted using LD pruning (Dudbridge and Newcombe 2015, Schmidt *et al.* 2020) and CHP bias-correction as described in the next section.

### 2.3 Handling linkage disequilibrium

**Figure 3.**
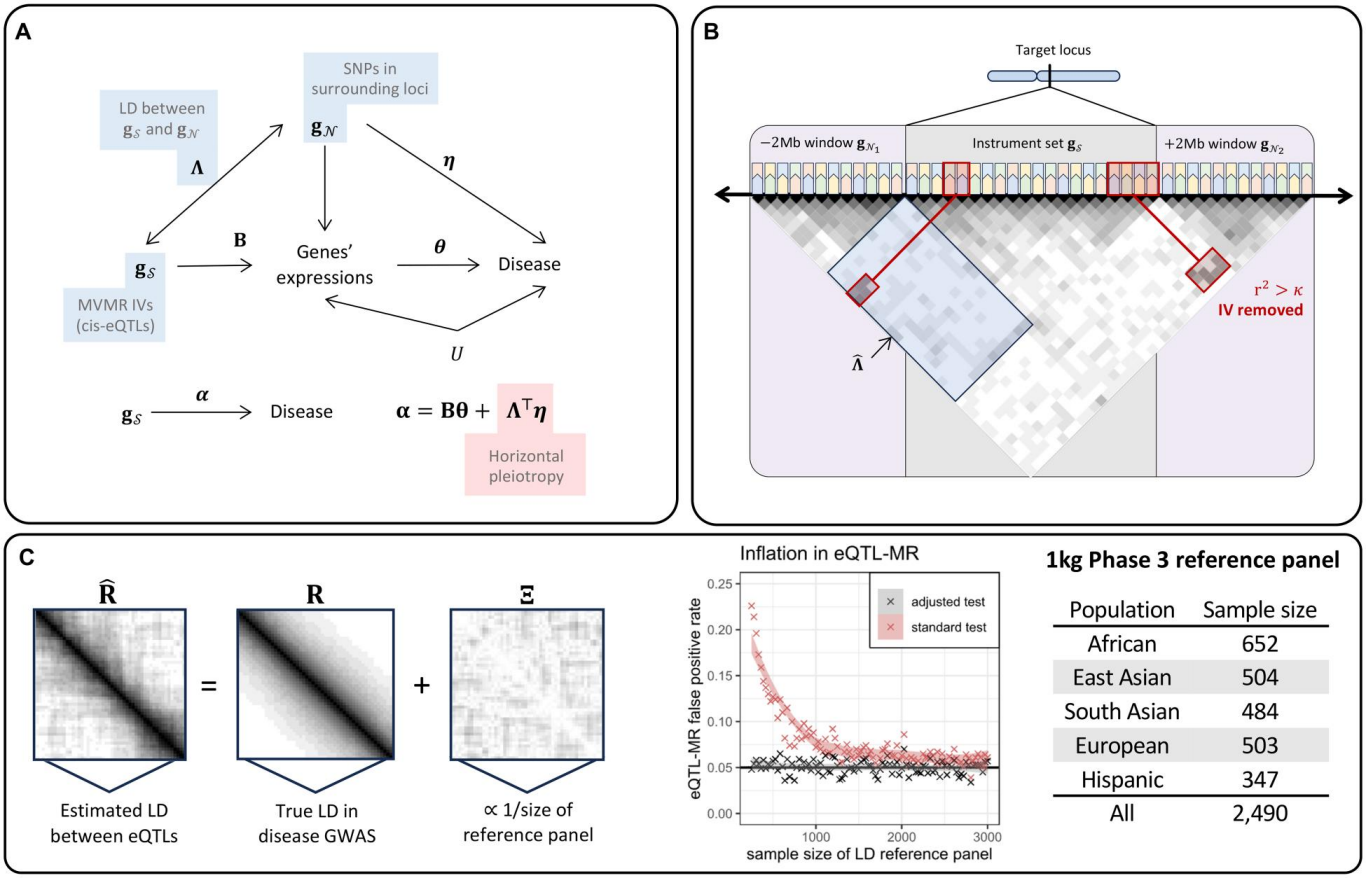
This figure illustrates the adjustments for CHP and inflation that are introduced when the eQTLs used in MR are in LD and researchers only have access to relatively small reference panels. (A) The goal of eQTL-MVMR is to estimate θ, which may be subject to bias when Λ and η are each nonzero. (B) This is the CHP-adjustment procedure described in Section 2.3.1. (C) Results in the panel entitled “Inflation in eQTL-MR” are from a simulation with 1000 replicates in which the true LD matrix had dimension 500×500 and a first-order autoregressive correlation structure with correlation parameter 0.5. We applied LD pruning at the threshold $r^{2}<0.3^{2}$. In this simulation, we repeatedly drew an estimate of the LD matrix from a Wishart distribution with degrees of freedom found on the *x*-axis. The R code used to perform this simulation is available at https://github.com/noahlorinczcomi/HORNET.

In nearly all applications of MVMR with eQTL data, an estimate of the LD matrix R for a set of eQTLs used as IVs is required. There are at least three primary challenges related to the use of eQTLs that are in LD when only individual-level data from a reference panel is available: (i) LD between causal SNPs can induce a CHP bias (see Supplementary Section S2.1), (ii) imprecise estimates of LD between the eQTLs can lead to underestimated standard errors of the causal effect estimates (Supplementary Sections S2.4 and S2.6), (iii) direct application of the estimated LD matrix to MR may be impossible because of non-positive definiteness and the choice(s) of regularization (Bickel and Levina 2008) may not always be clear. An additional challenge which HORNET does not address is the possibility of differences in the LD structure of the population used in GWAS and the LD reference panel. Figure 3 presents results from simulations demonstrating how this can affect inference using MR. In the next three subsections, we describe these challenges in greater detail and present the solutions that HORNET can implement.

#### 2.3.1 CHP from LD between eQTLs

CHP can be introduced in eQTL-MVMR if any eQTLs used as IVs in a target locus are in LD with other eQTLs that are not in the IV set. This is a form of confounding that can inflate Type I or II error rates when testing the causal null hypothesis (Morrison *et al.*, 2020; Verbanck *et al.*, 2018). We account for this CHP by removing IVs in the candidate set S that have LD $r^{2}>\kappa$ with other SNPs not in this set but within ±2 Mb of the boundaries of the locus. A visual example of this process is present in Fig. 3B. In practice, estimation of LD between eQTLs in the IV set and those outside of it is made using the available LD reference panel. This process will reduce the number of eQTLs available for use in MVMR, since it will remove IVs in LD with neighboring non-IVs, but may provide partial protection against CHP bias. As an example, we show in Supplementary Figs S10–S13 using Alzheimer’s disease that LD correlation between eQTLs in the IV set and those excluded from it introduced unbalanced horizontal pleiotropy bias for 5.2% of genes tested for causality on chromosome 19 using the IVW method. These results also suggested that multivariable IVW causal estimates statistically differed from univariable IVW causal estimates for 48.2% of genes (see Supplementary Section S2.1.3).

#### 2.3.2 Inflation from mis-specified LD

Mis-specifying the LD matrix corresponding to a set of eQTLs that are used as IVs in eQTL-MR can inflate the statistics used to test the causal null hypothesis (Jiang *et al.* 2022). Since individual-level data for the discovery GWAS of the disease phenotype are rarely publicly available, eQTL-MR relies on publicly available reference panels to estimate LD between a set of SNPs using populations which are assumed to be similar to the eQTL GWAS population. This LD matrix can be mis-specified when a reference panel of relatively small size and/or different genetic ancestry is used, making causal inference using standard MR methods such as IVW (Burgess and Bowden 2015) or principal components adjustment (Burgess *et al.* 2017) vulnerable to inflated Type I/II error rates (Jiang *et al.* 2022). No solution to this problem currently exists for eQTL-MVMR. We demonstrate in this section that this problem is caused by misspecification of the residual degrees of freedom in the standard *t*-test for statistical inference of a causal effect.

We therefore propose a *t*-test which is corrected for misspecification of the LD reference panel. Consider a univariable MR model using *m* IVs in which


$$\hat{\theta }=\left({\hat{\beta }}^{⊤}W^{-1}\hat{\alpha }\right)/\left({\hat{\beta }}^{⊤}W^{-1}\hat{\beta }\right)\hat{\alpha }∼N(\beta \theta ,R),\;W∼\mathrm{Wishar}t_{m}(n,n^{-1}R),$$

where *n* is the sample size of the LD reference panel. Standard practice to test $H_{0}:\theta =0$ compares $L=\hat{\theta }/\hat{\mathrm{SE}}(\hat{\theta })$ to a *t*-distribution with m−1 degrees of freedom. This test implicitly assumes that $\left(m-1)\hat{\mathrm{Var}}(\hat{\theta })/\mathrm{Var}(\hat{\theta })∼\chi^{2}(m-1\right)$, when in fact $\left(n-m+1)\hat{\mathrm{Var}}(\hat{\theta })/\mathrm{Var}(\hat{\theta })∼\chi^{2}(n-m+1\right)$ when W is treated as random (Mukhopadhyay 2009). The statistic L does not follow a *t*-distribution since the residual degrees of freedom is mis-specified. However, $\tilde{L}=\sqrt{\left(n-m+1\right)/n}L$ does follow a *t*-distribution with m−1 degrees of freedom. We therefore use the statistic $\tilde{L}$ to test $H_{0}:\theta =0$ instead of L. It follows from the definition of $\tilde{L}$ that $\tilde{L}\leq L$, which implies that it may be less powerful than L, but should also control Type I error rate at the nominal level. Figure 3 demonstrates that testing of the MR null hypothesis by current methods that use an estimated LD matrix is vulnerable to an inflated Type I error when the size of the LD reference panel is relatively small compared to the size of the IV set. Alternatively, the test introduced above which is based on a corrected degrees of freedom via the Wishart model maintains controlled Type I error at all sizes of LD reference panel. Supplementary Figs S19 and S20 compare the performance of the proposed inflation correction to that of standard MR methods including standard IVW, LD pruning, and principal components (Gkatzionis *et al.* 2023).

#### 2.3.3 Non-positive definite LD matrix

When using a reference panel to estimate LD between a set of eQTLs that may be used as IVs in eQTL-MVMR, the raw estimate $\hat{R}$ is not guaranteed to be positive definite, especially if the size of the reference panel *n* is less than the number of IVs (Gkatzionis *et al.* 2023). LD pruning also does not guarantee this issue will always be avoided. In this case, we may not be able to directly use $\hat{R}$ because eQTL-MVMR requires its inverse, which may not exist. Multiple solutions to this problem exist in the literature, with methods either transforming the IV set (Yang *et al.* 2012, Newcombe *et al.* 2016, Burgess *et al.* 2017) or directly applying regularization to $\hat{R}$ (Cheng *et al.* 2022). HORNET allows users to either prune the IV set using the PLINK clumping procedure (Purcell *et al.* 2007) or apply the scalar shrinkage method of Choi *et al.* (2019) to achieve a positive definite LD matrix with minimal perturbation. When pruning SNPs to achieve positive definiteness, users can define the LD threshold at which SNPs will be pruned, and the result of this procedure will generally reduce the number of eQTLs which are in the final IV set. This may reduce statistical power in MVMR if the heritability of gene expression is reduced by pruning away eQTLs with relatively large effect sizes. When users instead regularize the LD matrix by the method of Choi *et al.* (2019), the size of the IV set is left unchanged, but the working LD matrix is linearly shrunken by scalar factors toward the identity matrix in a minimal way such that it becomes positive definite.

### 2.4 Estimating causal effects

HORNET performs multivariable MR (MVMR) locus by locus across the genome. Standard causal inference from MVMR is based on the *P*-value corresponding to the estimated causal effect. We apply this inference and include two additional criteria to prioritize genes based on their significance and estimated causal effect size. These criteria are the (i) locus *R*-squared, measuring the total contribution of gene expression to phenotypic variation, and (ii) Pratt index (Aschard 2016). The HORNET software uses GScreen (see Section 2.4.1) to select genes for causal estimation and estimates causal effects using MRBEE (Lorincz-Comi *et al.* 2024), which we introduce briefly here. Let $\hat{B}={\left({\hat{\beta }}_{j}\right)}_{j=1}^{m}$ represent the m×p matrix of estimated SNP-expression associations for *m* SNPs and *p* genes in a single locus, selected such that each j∈S (Eq. (1)), and $\hat{\alpha }$ represent the m×1 vector of estimated SNP-phenotype associations. The MRBEE causal effects estimator is


(2) θ^=(B^⊤Rλ-1B^-mΣWβWβ)-1B^⊤Rλ-1α^

where $R_{\lambda }$ is a regularized LD matrix (see Section 2.3.3) and $\Sigma_{W_{\beta }W_{\beta }}$ the weak instrument bias matrix (see Algorithm 1). In practice, the IV set S is further restricted to only include SNPs which do not have evidence of horizontal pleiotropy using the IMRP procedure (Zhu *et al.* 2021b). Since MRBEE performs robust multiple regression, the corresponding variance explained R-squared values can be used to approximately represent the degree of model fit in a locus. We demonstrate in the Supplement that the locus R-squared is only equal to the true heritability explained when the power to detect each causal eQTL is 1. The Pratt index is gene-specific in a single locus and is used to represent the gene-specific proportion of variance explained in MVMR. Each locus will have one R-squared value and each gene in the locus will have its own Pratt index value, the sum of which across all genes in the locus is theoretically the locus R-squared value. We introduce the locus R-squared and gene-specific Pratt index values as imperfect measurements of quantities that are generally of interest when applying HORNET, and assert that the MVMR literature currently lacks any measurement which intends to capture what these two do.

#### 2.4.1 Screening genes

We stated in the previous section that each gene in a locus is first screened for evidence of causality then, if passing the screen, their causal effects are estimated using MRBEE. In this section, we briefly introduce the motivation for and execution of the screening process. In a locus of approximately 2 Mb, many genes may be present (e.g. upwards of 30). Given the restrictions placed on the structure of cis-eQTL data mentioned in Section 1, the curse of dimensionality may be frequently encountered, making direct estimation of all causal effects in a locus by MRBEE challenging. We therefore propose to first screen all genes in a locus using a variable selection penalty to reduce the dimensionality of MVMR (see Fan and Li 2001, Zhang 2010). This step will automatically select a relatively small subset of genes with the strongest evidence of direct causality of the outcome. We then apply MRBEE only to the selected genes passing this screening step. We use a new method called GScreen which approximates median regression using the methods of He *et al.* (2023) and applies the unbiased SCAD variable selection penalty (Fan and Li 2001) with hyper-parameters chosen as those which minimize a Bayesian information criterion (BIC) across a grid of candidate values. Supplementary Section S4 provides more details about the GScreen method and its performance in simulation and application to real data.

### 2.5 Simulations

We performed three separate simulations to assess the performance of missing data imputation, inflation in eQTL-MR, and inflation-correction methods, each of which used 1000 replicates. The setup of each simulation and a discussion of the results they produced are respectively described in the next three subsections. These simulations are intended to reflect the general scenarios described above related to missing in cis-eQTL effect sizes outside of the cis window and to causal inference from MVMR when the LD matrix is estimated imprecisely. The challenge of missingness in the candidate IV set is likely to be encountered in most applications of MVMR to eQTL summary data because of its inherent structure, and Type I error in MR will generally be inflated if multiple IVs are used and their LD structure is estimated imprecisely. These simulations are therefore intended to reflect general challenges of MVMR with cis-eQTL data, and not challenges related specifically to the application of HORNET.

#### 2.5.1 Imputing missing data

In the missing data simulation, we used summary statistics from eQTL GWAS for nine genes on chromosome 1 produced from 236 non-Hispanic White individuals. We restricted the eQTLs used to only those within ±2 Mb of the transcription start site (TSS) of one of the genes, producing 526 fully observed eQTLs. We then set the *Z*-statistics for eQTL-gene pairs in which the eQTL was >1 Mb from the TSS as missing and evaluated four methods of imputation: (i) MV-Imp, which was the matrix completion approach outlined in Algorithm 1, (ii) imputation of missing values with 0 s, (iii) soft impute (Mazumder *et al.* 2010), and (iv) imputation based on the multivariate normal distribution. For each simulation, the true LD correlation matrix R between the 526 eQTLs had a first-order autoregressive structure with correlation parameter 0.5. The matrix of measurement error correlations $\Sigma_{W_{\beta }W_{\beta }}$ was estimated from all SNPs in the 1 Mb window with squared *Z*-statistics for all eQTL associations less than the 95th quantile of a chi-square distribution with one degree of freedom. This follows the procedures used in practice (Zhu *et al.* 2015, Lorincz-Comi *et al.* 2024).

In simulation, our multivariate imputation method outlined in Algorithm 1 has smaller estimation error than imputation with all zero values or the traditional soft impute method (Mazumder *et al.* 2010). Estimation error in this setting is defined as the difference between true and imputed values. Since there is currently no other way to address missing data in eQTL-MVMR, zero imputation, soft impute, and imputation based on the multivariate normal distribution are three straightforward alternatives to our proposed imputation approach. We demonstrate in Supplementary Section S1.4 and Fig. 2B that imputing missing data using our algorithm can produce up to 2–4× increases in power versus excluding eQTLs with any missing associations as IVs. However, as Supplementary Section S6 shows using real data, imputing missing eQTL effect sizes using our imputation approach may not always lead to better fitting models compared to imputation with zeros.

#### 2.5.2 Inflation in eQTL-MR

In the simulation to demonstrate inflation in eQTL-MR, the true LD matrix R for 500 eQTLs had a first-order autoregressive structure with correlation parameter 0.50 and was estimated by sampling from a Wishart distribution with varying degrees of freedom equal to the reference panel sample size. In each simulation, true eQTL and disease standardized effect sizes were drawn from independent multivariate normal distributions with means 0 and covariance matrices R. We then applied LD pruning (Dudbridge and Newcombe 2015, Schmidt *et al.* 2020) at the threshold $r^{2}<0.3^{2}$ to restrict the IV set used in univariable MR. We performed MR using univariable IVW (Burgess and Bowden 2015) and Type I error rate was recorded using both the standard test statistic L and the adjusted statistics $\tilde{L}$ introduced in Section 2.3.2. Type I error rate was based on tests of the causal null hypothesis.

Figure 3C demonstrates that LD reference panels that contained genotype information for less than 3000 individuals inflated the false positive rate in eQTL-MVMR using the standard test statistic *L*. When the reference panel contained 500 individuals, the false positive rate approached 0.25 using *L*. As a comparison, the largest population-stratified sample of individuals in the 1000 Genomes Phase 3 reference sample (The 1000 Genomes Project Consortium *et al.* 2015) is 652 and the smallest is 347. Using our adjusted test statistic $\tilde{L}$, Type I error rate was controlled at the nominal level for LD reference panels of any size, providing support that this method of hypothesis testing may not have inflated Type I error. As mentioned above, our adjustment for inflation in eQTL-MVMR from imprecisely estimated LD structure between eQTLs relies on the assumption that the inverse of their LD matrix has population moments which are consistent with the Wishart distribution. Since the Wishart distribution is a natural distribution from which sample covariance matrices can be generated, this assumption was implicitly met in our simulations.

### 2.6 Software

HORNET requires GWAS summary statistics for gene expression and a disease phenotype and an LD reference panel. LD estimation from a reference panel for a set of eQTLs is made using the PLINK software (Purcell *et al.* 2007), which requires the presence of .bim, .bed, and .fam files. eQTL GWAS data must contain a single file for each chromosome and generally should contain summary statistics for all genotyped SNPs within a cis-region of each available gene. These data are available for blood tissue from the eQTLGen Consortium (*n* = 31k) (Vosa *et al.* 2018) and the GTEx consortium for 53 other tissues (*n* *<* 706) (2015). To help researchers identify relevant tissues to select in their analyses, we provide a tissue prioritizing tool based on the heritability of eQTL signals. This tool receives a list of target genes from the researcher and returns a ranked list of tissues in which each target gene has the strongest eQTLs using GTEx v8 summary data. See Supplementary Section S4 for additional details and a demonstration of how to use this tool. HORNET spends approximately 7.5 min per chromosome on average when using its default settings of IV selection and network construction, and its average run time is not affected by the choice of missing data imputation. More details regarding its run time and performance in under various combinations of its main parameters are presented in Supplementary Section S6. Since current MVMR methods intended for use with eQTLs generally apply standard MVMR estimators to eQTL data while incorporating an LD matrix, the parameters of HORNET can be set such that standard eQTL-MVMR methods are used, which are shown in Supplementary Section S6.

The HORNET software exists as a command line program available for Linux, Windows, and Mac machines. Its tutorial is available at https://github. com/noahlorinczcomi/HORNET and is introduced briefly in Supplementary Section S5. By downloading HORNET, users also receive PLINK v1.9 (Purcell *et al.* 2007) and LD reference panels for European, African, East and South Asian, Hispanic, and trans-ethnic populations from 1000 Genomes Phase 3 (1 kg) (The 1000 Genomes Project Consortium *et al.* 2015). By default, our software uses this reference panel from the entire 1 kg sample to estimate LD in the eQTL GWAS population, but users can alternatively specify a specific sub-population in 1 kg or even use their own LD reference panels.

## 3 Results

### 3.1 Real data analysis with schizophrenia

**Figure 4.**
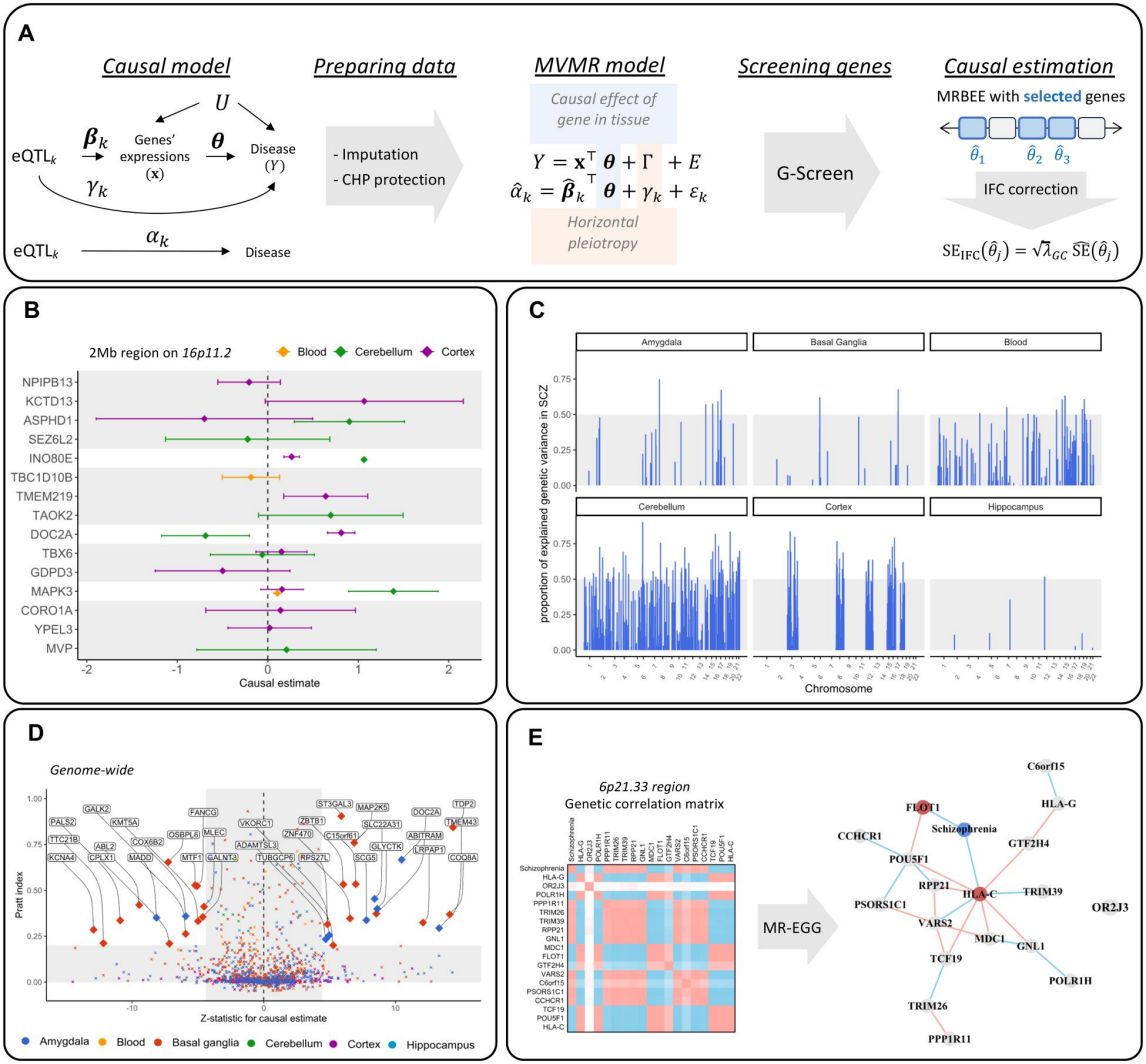
This figure presents the results of using HORNET to search for genes modifying schizophrenia risk when expressed in different tissues. (A) Description of the causal model, MVMR model, and estimator. (B) Causal estimates for multiple genes in blood, cerebellum, and cortex tissues in the schizophrenia-associated *KCTD13* locus. (C) *R*-squared values from MVMR models fitted across the genome. Areas in which no *R*-squared values exist either had no genes prioritized by GScreen or had insufficient eQTL signals to perform MVMR. (D) Pratt index values for all causal estimates made for all tissues. Pratt index values outside the range of (−0.1, 1) are not shown. This may happen because of large variability in univariable MR estimates for some loci. (E) Estimated gene co-regulatory between genes in the FLOT1/HLA-C locus and including schizophrenia using the MR-EGG approach (Yang *et al.* 2024).

We applied the HORNET methods and software to the study of genes whose expression in basal ganglia, cerebellum, cortex, hippocampus, amygdala, or blood tissue may cause schizophrenia risk using summary eQTL and schizophrenia GWAS data. Schizophrenia GWAS data were from Trubetskoy *et al.* (2022), which included 130k European individuals and were primarily from the Psychiatric Genomics Consortium (PGC) core dataset. eQTL summary statistics in brain tissue were from de Klein *et al.* (2023), which contained data from European samples of sizes 208 for basal ganglia, 492 for cerebellum, 2683 for cortex, 168 for hippocampus, and 86 for amygdala tissue. eQTL GWAS data in blood were from the eQTLGen Consortium (Vosa *et al.* 2018) for 31k predominantly European individuals. We performed analyses with HORNET in all schizophrenia loci with at least one *P*-value in the schizophrenia GWAS that was less than .005. This heuristic threshold was set to be high enough so that many loci would be analyzed using HORNET, but low enough so that loci containing absolutely no evidence of association with SCZ were not analyzed. We grouped genes that shared eQTLs with *P*-values less than .001, applied LD pruning to these eQTLs at the threshold $r^{2}<0.7^{2}$, and removed SNPs in LD with any IVs in the target locus beyond $r^{2}>0.5^{2}$ in a 1 Mb window. The nominal *P*-value threshold for eQTLs was set to be relatively permissive to account for the reduced power of the intersection union test which was effectively performed across all genes in the locus (Berger and Hsu 1996). All IVs had a *P*-value for joint association with gene expression across all tissues which was less than $5\times 10^{-3}$ in the test of Equation (1). Inclusion of eQTLs not truly associated with any tested gene are expected to reduce the power of causal effect hypothesis testing, but not to bias the causal effect estimate since this bias is corrected for by the MRBEE estimator (Lorincz-Comi *et al.* 2024). We performed HORNET in each tissue separately and present the results in Fig. 4.

Figure 4 uses the data described above to provide examples of the primary results produced by genome-wide analysis with HORNET, including causal estimates for prioritized genes, genome-wide R-squared, and Pratt index values for each tissue, and an estimated sparse co-expression network of genetic correlations using MR-EGG (Yang *et al.* 2024). These results show that locus *R*-squared values can exceed 0.50 for many loci, suggesting that SNP associations with schizophrenia in these loci may be primarily explained by gene expression in brain tissue (Panel c). For example, 17.2% of genetic variation in schizophrenia in the *KCTD13* locus is explained by the expression of genes in blood tissue, 75.2% in the cerebellum, and 59.4% in the cortex. In this locus, *DOC2A* eQTL *Z*-statistics in cortex tissue (GTEx v8) are moderately correlated with schizophrenia *Z*-statistics (Pearson *r* = 0.52), although the lead *DOC2A* eQTL (rs35695082) only has a *P*-value of .013. Similarly, in cerebellum tissue (GTEx v8), *DOC2A* eQTL *Z*-statistics are moderately correlated with schizophrenia *Z*-statistics (*r* = −0.52), though the lead eQTL (rs35695082) only has *P*-value of .001. Alternatively, expression of the *INO80E* in cerebellum tissue (GTEx v8) was associated with increased schizophrenia risk, and in the data we used in this study, the lead *INO80E* eQTL in cerebellum tissue (rs3814881; *P* = $2\times 10^{-14}$) had a *P*-value of $4.2\times 10^{-14}$ in the schizophrenia GWAS, provider greater evidence of a potential functional mechanism than in the case of *DOC2A*.

*INO80E* is in a gene-dense region of chromosome 16 (16p11.2) containing multiple genes that have evidence of association with schizophrenia (Ikeda *et al.* 2019), but has historically been of particular interest to researchers in the context of schizophrenia because of its role in DNA damage repair and neurotransmitter release (Poli *et al.* 2017). *INO80E* has evidence of association with schizophrenia in SNP-based (Ikeda *et al.* 2019) and gene-based association testing (Lorincz-Comi *et al.* 2025), and from transcriptome-wide association and MR studies (Liu *et al.* 2020, Chang *et al.* 2021, Uellendahl-Werth *et al.* 2022, Liufu *et al.* 2024). Copy number variations in the *INO80E* region have also been shown to be enriched in schizophrenia cases (Glessner *et al.* 2010), and a region of open chromatin in neurons from brain tissue in the region of *INO80E* may imply a functional relationship with schizophrenia pathology (Dang *et al.* 2025). Together, these results provide additional support for a potentially causal relationship between expression of *INO80E* and schizophrenia risk, though additional contexts such as RNA splicing or protein abundance should also be explored to better understand its role in pathology and as a potential drug target.

We attempted to better understand the complex co-expression network that may exist between genes in the human leukocyte antigen (HLA) complex of 6p21.33 (Klein and Sato 2000). Genetic variants in this region are associated with risk of schizophrenia (SPGWAS Consortium 2011, Goes *et al.* 2015, Ikeda *et al.* 2019) and many other traits such as brain morphology (Chen *et al.* 2020), autism spectrum disorder (Anney *et al.* 2017), and Type II diabetes (Vujkovic *et al.* 2020). The HORNET software applied MR-EGG (Yang *et al.* 2024) to the matrix of imputed marginal eQTL *Z*-statistics to estimate undirected relationships between 18 genes in this locus and their pathways of putative causal effect on schizophrenia risk when expressed in cerebellum tissue. Nodes in this network include 18 genes and schizophrenia, and MR-EGG estimates the sparse inverse of the matrix of robust correlations between SNP effect sizes on gene expression and schizophrenia to infer nonzero conditional dependence between nodes. For example, an edge connecting two genes is inferred to represent a correlation between cis-eQTLs for those genes which is not explained by their eQTL correlations with other traits in the network. Sparsity on the co-expression network is imposed by searching a grid of penalizing hyper-parameters and selecting those which minimize a BIC to produce the final network (see Yang *et al.* (2024) for additional details). The results of these analyses suggest a connected gene co-expression network in which the *HLA-C* gene may be a regulatory hub (Yu *et al.* 2017, Deng *et al.* 2018). eQTLs for the *HLA-C* gene are directly associated with the eQTLs of eight other genes and are indirectly associated with the eQTLs of all genes in the locus except *OR2J3*. Only *HLA-C* and *FLOT1* are inferred to have direct causal effects on schizophrenia risk, and all other 15 peripheral genes (*OR2J3* excluded) may have causal effects on schizophrenia that only are mediated by *FLOT1* and/or *HLA-C* expression.

## 4 Discussion

Existing multivariable MR methods using GWAS summary statistics may be vulnerable to bias and inflation from missing data, mis-specified LD structure, and confounding by other genes when directly applied to cis-eQTLs. Equally, no flexible and comprehensive set of computational tools to robustly perform this task current exists. We introduced a suite of statistical and computational tools in the HORNET software that addresses these common challenges in multivariable cisMR using eQTL GWAS data. HORNET is currently restricted to cis-eQTLs since is the primary mode of transcriptomic MR analysis practiced in the literature, though future versions of HORNET may allow users to include both cis and trans eQTLs as IVs in MVMR. Where the trans eQTL data are available to researchers, there may be less need for imputation approaches such as those we have outlined above, and the potential for CHP bias from LD-correlated eQTLs that are omitted from the cis-eQTL IV set may be lower. HORNET can generally provide unbiased causal estimation and robust inference across a range of real-world conditions in which existing methods in alternative software packages may not. HORNET is a command line tool that can be downloaded from https://github.com/noahlorinczcomi/HORNET, where users will also find detailed tutorials demonstrating how to use HORNET.

## Supplementary Material

vbaf068_Supplementary_Data

## Data Availability

The data underlying this article are available at https://github.com/noahlorinczcomi/HORNET. Algorithm 1. Pseudo-code of eQTL imputation
**Require:** The m×p incomplete matrix of eQTL association estimates between m SNPs and expressions of p genes $\hat{B}$, the set of missing values O, the singular values $\eta_{1}\geq \ldots \geq \eta_{p}$ of the p×p weak instrument bias matrix $m\Sigma_{W_{\beta }W_{\beta }}$, inverse LD matrix Θ, tuning parameter λ, tolerance ϵ.   1. Initialize ${\hat{B}}^{0}=\Theta^{1/2}\hat{B}$ with missing values set to 0   2. Define $d_{1}^{0}\geq \ldots \geq d_{p}^{0}$ as the singular values of ${\hat{B}}^{0}≔\mathrm{UD}V^{⊤}$   3. Define $\alpha =1-\sum_{k=1}^{p}\eta_{k}/\sum_{k=1}^{p}d_{k}^{0}$   4. Reconstruct ${\hat{B}}^{0}=U(\alpha D)V^{⊤}$, where $D=\mathrm{diag}{\left(\alpha \times d_{k}^{0}\right)}_{k=1}^{p}$   while do ${\left\|{\hat{B}}^{\left(t+1\right)}-{\hat{B}}^{\left(t\right)}\right\|}_{F}>\epsilon$    Find $\mathrm{UD}V^{⊤}={\hat{B}}^{\left(t\right)}$ and define the kth singular value as $d_{k}^{\left(t\right)}$    Threshold singular values, $d_{k}^{\left(t+1\right)}={\left(d_{k}^{\left(t\right)}-\lambda \right)}_{+}$; where ${\left(a\right)}_{+}=\max\left(0,a\right)$    Construct ${\hat{B}}^{\left(t+1\right)}=UD^{+}V^{⊤}$, where $D^{+}=\mathrm{diag}{\left[d_{k}^{\left(t+1\right)}\right]}_{k=1}^{p}$    Set ${\hat{B}}_{/O\;}^{\left(t+1\right)}={\hat{B}}_{/O\;}^{\left(t\right)}$, i.e. only missing values are imputed   **end while**
**Ensure:** Matrix ${\hat{\Theta }}^{-1/2}{\hat{B}}^{\left(t\right)}$ with no missing values
